# Thermoreversible (Ionic-Liquid-Based) Aqueous Biphasic Systems

**DOI:** 10.1038/srep20276

**Published:** 2016-02-04

**Authors:** Helena Passos, Andreia Luís, João A. P. Coutinho, Mara G. Freire

**Affiliations:** 1 CICECO - Aveiro Institute of Materials, Department of Chemistry, University of Aveiro, 3810-193 Aveiro, Portugal

## Abstract

The ability to induce reversible phase transitions between homogeneous solutions and biphasic liquid-liquid systems, at pre-defined and suitable operating temperatures, is of crucial relevance in the design of separation processes. Ionic-liquid-based aqueous biphasic systems (IL-based ABS) have demonstrated superior performance as alternative extraction platforms, and their thermoreversible behaviour is here disclosed by the use of protic ILs. The applicability of the temperature-induced phase switching is further demonstrated with the complete extraction of two value-added proteins, achieved in a single-step. It is shown that these temperature-induced mono(bi)phasic systems are significantly more versatile than classical liquid-liquid systems which are constrained by their critical temperatures. IL-based ABS allow to work in a wide range of temperatures and compositions which can be tailored to fit the requirements of a given separation process.

Liquid-liquid extraction processes are technologically simple, of low cost and often highly effective for the separation of a large plethora of compounds or materials. Consequently, liquid-liquid extraction has regularly been a favoured choice in process engineering. These systems can display a strong temperature dependence and be used as switchable systems, as attempted by Bergbreiter *et al.*^1^, who proposed the use of thermomorphic catalysis, which allows both the product separation and the catalyst recovery. Other organic-organic or organic-aqueous systems have been used in the separation of catalysts, metals, organic and biological products^2^. However, the use of volatile and hazardous organic solvents in extraction processes presents major drawbacks. In this context, ionic liquids (ILs) represent a viable alternative due to their non-volatile nature coupled to a high and tailored solvation ability.

In the past few years, the research on dynamic and reversible biphasic systems constituted by ILs has received a crucial attention towards the development of novel and more efficient separation processes^3^,^4^,^5^. It was demonstrated that phase transitions in mixtures involving ILs and other solvents can be induced by changes in temperature or by reversible reactions with CO_2_^6^,^7^,^8^,^9^,^10^,^11^,^12^,^13^,^14^,^15^. Some IL/solvent mixtures display an upper critical solution temperature (UCST)^16^ whereas others present a lower critical solution temperature (LCST)^17^. These temperature-dependent phase transitions have shown to be highly advantageous in the selective separation of proteins^18^, metals^16^ and catalysts^1^,^9^. However, most UCST and LCST in IL-containing systems often occur at temperatures away from room temperature and are confined to mixture compositions imposed by the critical point of each phase diagram. Therefore, the design of novel systems with UCST or LCST close to room temperature has been object of a great deal of work; yet, only a restricted number of systems has been identified^3^,^8^,^17^. Moreover, these systems are composed of an IL-rich phase (typically with hydrophobic characteristics) and a molecular-solvent-rich phase^16^,^17^. Reversible liquid-liquid systems have also been prepared with molecular solvents that react with CO_2_ forming salts and/or ILs^6^,^7^,^8^,^19^. Nevertheless, this type of reversibility requires the flushing of the system with gases and the use of specific equipment^6^,^7^,^8^.

The research in liquid-liquid extractions using ILs focuses on two main approaches: (*i*) the direct use of hydrophobic ILs^11^,^12^,^18^,^20^ which leads to the formation of an IL- and a water- or organic-solvent-rich phase (where the UCST and LCST reversible temperature-induced systems fall); and (*ii*) the use of aqueous biphasic systems (ABS) composed of ILs and organic/inorganic salts^21^,^22^ that above given concentrations lead to the formation of two aqueous-rich phases. ABS are commonly seen as “greener” and more biocompatible options since they are mostly composed of water (up to 70 wt % in the overall system)^22^. Furthermore, one of the most outstanding features of ILs - their tailoring capacity by appropriate cation/anion combinations - is transposable to IL-based ABS, and thus, these systems allow an efficient design for the selective extraction of a variety of compounds^21^,^22^,^23^,^24^.

The number of potential IL-based liquid-liquid extraction routes surpasses by far conventional polymer-salt or polymer-polymer ABS^21^,^22^. Typical ABS have been largely investigated in the extraction and purification of proteins, including value-added antibodies, cells organelles and viruses^25^,^26^. Nevertheless, these systems display restricted differences on their phases’ polarities and affinities for target compounds which have been obstructing high selectivities and extraction efficiencies to be attained. Due to their tailoring ability, IL-based ABS have shown to be promising extraction/purification routes for a large plethora of biologically active compounds, *e.g.* proteins, enzymes and biopharmaceuticals^22^,^27^,^28^,^29^,^30^,^31^,^32^. In most studies, the complete extraction of the biological compounds was attained in a single-step without denaturation or precipitation effects^29^,^30^,^31^. Moreover, the application of IL-based ABS to real matrices, *e.g.* bovine serum to extract bovine serum albumin^33^, and the recovery and reuse of the IL-rich phase was also demonstrated^29^. Nevertheless, imidazolium-28, cholinium-33 and tetralkylphosphonium-based ILs^29^ have been the preferred choice as phase-forming components of ABS and no studies comprising protic ILs have been found.

In addition to the well-established outstanding extraction performances of IL-based ABS, their temperature dependent phase-behaviour is a relevant aspect, though still scarcely explored^21^,^22^. Most studies were carried out at a fixed temperature since only ABS formed by aprotic ILs combined with salts or polymers were investigated, and these display a weak dependence on temperature^21^,^22^.

Herein, we reveal a novel class of thermoreversible ABS formed by protic ILs (PILs) and polymers, in particular poly(propylene) glycol (PPG) with an average molecular weight of 400 g∙mol^–1^. PILs are formed in one-step reactions between a low cost acid, such as acetic acid, and a base (*e.g.*, an amine)^34^. Their easy synthesis is a major advantage that coupled to their lower cost and more benign character^34^, make of them viable candidates to replace the aprotic ILs used in ABS formulations. The biocompatible nature of PPG 400 is also well-established^30^. As shown below, the phase diagrams of PIL-based ABS are highly dependent on temperature, with small changes on temperature being enough to trigger the phase transition.

## Results

### Ionic-liquid-based aqueous biphasic systems at different temperatures

**Figure 1:**
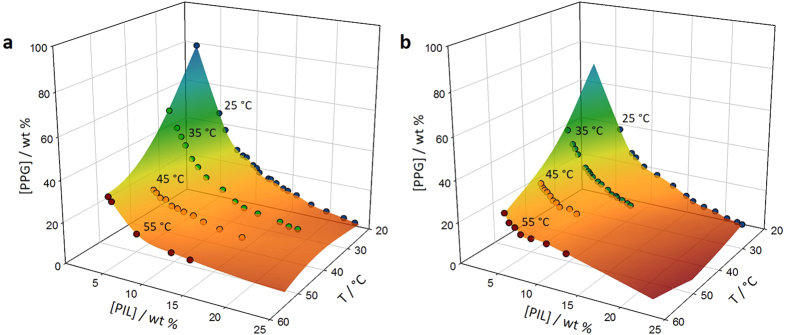
3D representation of the temperature effect in the IL-based ABS. Ternary phase diagrams for (**a**) [N_1120_][C_1_CO_2_] + PPG + H_2_O and (**b**) [N_11[2(N11)]0_][C_1_CO_2_] + PPG + H_2_O systems at different temperatures.

To study the thermoreversibility of PIL-based ABS, the respective phase diagrams at four temperatures (25, 35, 45 and 55 °C), for the systems composed of water, PPG and six PILs - *N,N*-dimethyl-*N*-ethylammonium acetate, [N_1120_][C_1_CO_2_]; *N,N*-diethyl-*N-*methylammonium methane sulfonate, [N_1220_][C_1_SO_3_]; *N*,*N*-dimethyl-*N*-(*N*´,*N*´dimethylaminoethyl)ammonium acetate, [N_11[2(N110)]0_][C_1_CO_2_]; *N*,*N*-dimethyl-*N*-(*N*´,*N*´dimethylaminoethyl) ammonium chloride, [N_11[2(N110)]0_]Cl; *N,N*-dimethyl-*N*-ethylammonium phenylacetate, [N_1120_][C_7_H_7_CO_2_]; and *N,N*-dimethyl-*N*-(*N*´,*N*´dimethylaminoethyl) ammonium octanoate, [N_11[2(N110)]0_][C_7_CO_2_] - were determined. ^1^H and ^13^C NMR spectra of pure PILs and of the water-rich phases after being submitted to 55 °C are presented in Supplementary Figs 1–11. Two examples of their liquid-liquid phase diagrams are depicted in Fig. 1. The detailed experimental weight fraction data, as well as the representation of the phase diagrams for the remaining ILs are presented in the Supplementary Tables 1–4 and Supplementary Fig. 12, respectively, along with the compositions of the coexisting phases, *i.e.*, the respective tie-lines and tie-line lengths (Supplementary Table 5).

For all phase diagrams, the biphasic region is located above the binodal curve while the monophasic region is localized below. In general, the larger the biphasic region, the higher is the capacity of the system to undergo liquid-liquid demixing. For a fixed temperature, *e.g.,* 25 °C, and at 20 wt % of PPG, the PILs capability to form ABS follows the order: [N_1220_][C_1_SO_3_] < [N_1120_][C_1_CO_2_] ~ [N_11[2(N11)]0_][C_1_CO_2_] < [N_11[2(N11)]0_]Cl – *cf.* the Supplementary Fig. 13. Amongst the studied ILs, [N_1120_][C_7_H_7_CO_2_] and [N_11[2(N11)]0_][C_7_CO_2_] were not able to induce ABS formation at any of the temperatures investigated. The presence of an aromatic ring and a long alkyl chain length at the anion and cation of [N_1120_][C_7_H_7_CO_2_] and [N_11[2(N11)]0_][C_7_CO_2_], respectively, increase the hydrophobicity of these PILs further preventing the formation of ABS with PPG. In summary, PILs constituted by more hydrophilic anions, *i.e*., anions with higher affinity for water, are more favourable for ABS formation. These trends are in agreement with previous works^35^, for which the higher the IL ion’s ability to create hydration complexes the more easy is the formation of IL-polymer ABS. It should be highlighted that the differences between the binodal curves for the various ILs investigated are more significant at higher temperatures.

In the studied ABS at 25 °C, the top phase corresponds to the PPG-rich phase, while the bottom phase is enriched in PIL and water, with the exception of the [C_1_CO_2_]-based PILs for which the opposite behavior was observed. Curiously, at 45 °C it was observed an inversion on the phases’ densities for the [N_11[2(N11)]0_][C_1_CO_2_]-based ABS, where the PIL-rich phase corresponds to the bottom layer as confirmed by conductivity measurements presented in Supplementary Table 6.

Figure 1 shows the effect of temperature on the ternary phase diagrams of the systems composed of [N_1120_][C_1_CO_2_] + PPG + H_2_O and [N_11[2(N11)]0_][C_1_CO_2_] + PPG + H_2_O. The depicted surfaces represent the limit between the monophasic and biphasic regions revealing that a temperature increase enhances the ability of PILs to form ABS - at higher temperatures lower amounts of IL or PPG are required for phase demixing. Albeit the phase diagrams depicted in Fig. 1 are presented at intervals of 10 °C, it should be remarked that the differences observed are large enough to trigger the reversible behaviour by changes in temperature as small as 1 °C.

### Thermoreversible behaviour

**Figure 2:**
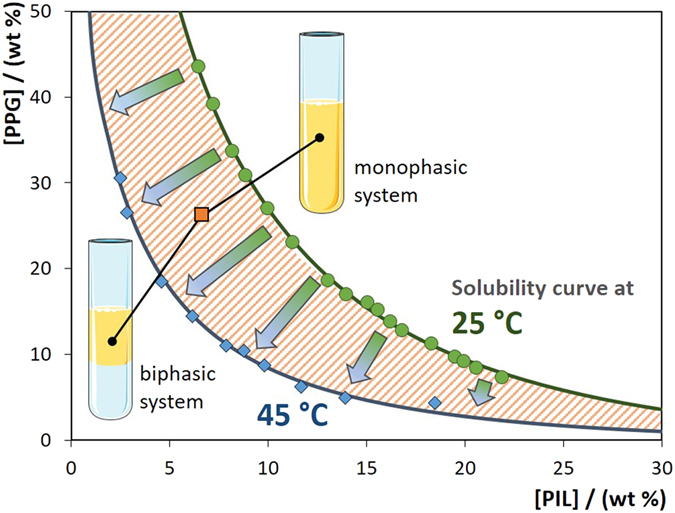
Schematic representation of PIL-based ABS thermoreversibility. Binodal curve of the ternary system composed of [N_11[2(N11)]0_]Cl + PPG + H_2_O. Green circles: 25 °C; blue diamond: 45 °C; orange square: initial mixture composition.

Upon the establishment of the temperature dependency of the studied ABS, their reversible behaviour was further ascertained. For that purpose, a monophasic ternary mixture was prepared at 25 °C, with a composition within the hatched region of Fig. 2, and the temperature was then increased to 45 °C resulting in the phase separation. As the systems is cooled down to 25 °C the system becomes monophasic again. This reversible behaviour can be applied as many times as desired without changes in the composition of the coexisting phases for a given initial mixture. Even so, one of the major advantages of these reversible IL-based ABS is that the temperature range of operation can be selected based on the ternary mixture composition to fit the requirements of a specific process (stability of the biomolecule being purified or optimization of the extraction efficiencies) and is not restricted to fixed temperatures imposed by the thermodynamic nature of binary liquid-liquid systems (UCST or LCST)^16^,^17^,^36^,^37^,^38^. Surfactant-based aqueous two-phase systems are also thermoreversible systems and have been extensively explored in the past decades for (bio)separation approaches^39^. Nevertheless, mostly aqueous two-phase micellar systems are binary systems, and thus, only one solubility curve exists, *i.e.*, for a given concentration of surfactant, a given temperature has to be reached to allow the liquid-liquid demixing. In ABS, while being ternary systems, the temperature at which the phase separation is carried out can be tuned by a simple manipulation of the concentration of the phase-forming components. In summary, thermoreversible IL-based ABS allow the design of their operating temperatures by defining the mixture compositions, resulting thus on more flexible and tailored liquid-liquid extraction processes.

### Applicability of protic-ionic-liquid-based aqueous biphasic systems

The applicability of the investigated thermoreversible IL-based ABS was further evaluated for separation processes, using two added-value proteins, namely cytochrome c and azocasein. For this purpose, homogeneous ternary mixtures composed of 6 wt % of [N_11[2(N11)]0_][C_1_CO_2_] + 30 wt % of PPG + 64 wt % of aqueous solutions containing the proteins at 1, 2 and 3 g·L^-1^ were prepared at 25 °C. Certainly, other points could be selected within this region and which allow the reversible cycles among monophasic and biphasic regimes to be achieved, by simple changes in temperature. Even so, a point using a small amount of IL was selected while attempting the demonstration of low-cost and biocompatible thermoreversible ABS. The temperature was then increased up to 45 °C to induce the phase separation and the proteins partitioning between the coexisting phases.

**Figure 3:**
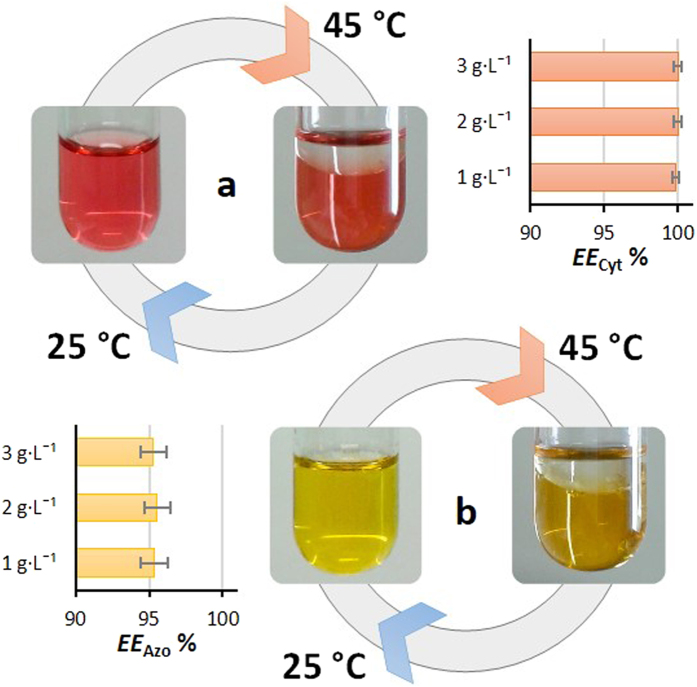
Partitioning of cytochrome c and azocasein at three different concentrations (1, 2 and 3 g·L^−1^) in PIL-based ABS formed at 45 °C. Extraction efficiency of (**a**) cytochrome c (*EE*_Cyt_%) (**b**) azocasein (*EE*_Azo_%). The extraction efficiency data are presented in Supplementary Table 7.

Remarkably, the two proteins, and at the three concentrations investigated, almost completely migrate for the PIL-aqueous-rich phase in a single-step (Fig. 3). The coexisting phases of the [N_11[2(N11)]0_][C_1_CO_2_]-based ABS display a pH value of circa 7.9 (see Supplementary Table 8), and according to the proteins isoelectric points (10.2 for cytochrome c^18^ and 4.8 for casein^40^), both macromolecules are charged - cytochrome c is positively charged while azocasein is negatively charged – and both preferentially partition for the phase with the higher ionic strength. Moreover, the PIL-rich phase also corresponds to the most “hydrated” layer – higher water content as revealed by the tie-lines data shown in the Supplementary Table 5. Pure ILs have almost no ability to solubilize proteins without denaturation^41^. In these, the modification of proteins is usually carried out with amphiphilic polymers^42^. The chemical modification involves multi-step treatments and further purification steps, which may cause the denaturation of proteins. However, some “hydrated ILs” or ILs aqueous solutions have demonstrated to be extraordinary media for solubilizing and stabilizing proteins^18^. LCST-type phase diagrams of IL-water mixtures were previously proposed by Ohno and co-workers^3^,^18^ for the separation of proteins. These systems are mostly composed of an IL-rich phase, for which the proteins partition, and an almost pure water phase. Nevertheless, the authors^18^ demonstrated that cytochrome c was not extracted into the IL-phase for water contents below 13 wt %. In this work, taking advantage of two aqueous-rich phases formed by ternary mixtures, in particular of an IL-rich phase with high amounts of water that can be properly tuned by variations either in the initial mixture composition or in temperature, the complete extraction of proteins in a single-step was observed. Furthermore, the cyclic reversibility between monophasic and biphasic regimes, driven by a decrease/increase in temperature, was demonstrated with 3 cooling-heating cycles, with no losses on the extraction efficiencies obtained for the two proteins. Although several promising approaches have been proposed for the recovery of the most diverse value-added compounds from the IL-rich phase^43^,^44^,^45^, the recovery of both proteins from the PIL-rich phase can be simple attained by a dialysis process^29^.

The proteins stability at 45 °C in the PIL-rich phase was also ascertained by FTIR spectroscopy. The proteins secondary structure can be studied through the analyses of amine I and II bands. The amide I band represents primarily the C = O stretching vibration of the amide groups and occurs near 1650 cm^−1^, while the amide II band represents the C─N stretching vibrations and occurs close to 1550 cm^−1^ 46. Through the results shown in Supplementary Figs 14 and 15 it is possible to conclude that both proteins maintain their native structures in the PIL-rich phase after the extraction being conducted at 45 °C. Fujita *et al.*^47^ and Ohno and co-workers^18^,^48^ reported that hydrophilic ILs having carboxyl residues are effective for dissolving and maintaining the structure of proteins. In this context, the PILs derived from carboxylic acids used in this work seem to be an appropriate option to form ABS envisaging their use in downstream processes, namely on the extraction and purification of proteins, enzymes and antibodies.

In summary, the results here presented disclose that IL-based ABS can be highly temperature dependent, allowing to trigger reversible phase separations by small changes in temperature. Furthermore, the working temperature range is not restricted to fixed temperatures imposed by the thermodynamic nature of binary liquid-liquid systems. Instead, not only the phase behaviour but also the water content and extraction performance of IL-based ABS could be controlled by selecting a suitable triad (nature of the phase-forming components, mixture compositions and temperature). The temperature-driven reversible behaviour of IL-based ABS allows them to be used as novel separation platforms and boosts the potential applicability of ILs in biomedical and pharmaceutical fields.

## Methods

### Materials

The determination of the liquid-liquid ternary phase diagrams was performed using aqueous solutions of PPG 400 (from Sigma-Aldrich) and individual aqueous solutions of the following PILs: [N_1120_][C_1_CO_2_] (98 wt %), [N_1220_][C_1_SO_3_] (>97 wt %), [N_11[2(N11)]0_][C_1_CO_2_] (98 wt %), [N_11[2(N11)]0_]Cl (97 wt %), [N_1120_][C_7_H_7_CO_2_] (>98 wt %), and [N_11[2(N11)]0_][C_7_CO_2_] (>98 wt %). All ammonium-based ILs were purchased from Iolitec and their chemical structures are shown in Supplementary Fig. 1. To reduce the volatile impurities to negligible values, PIL individual samples were purified under constant agitation, vacuum, and at moderate temperature (50 °C), for a minimum of 24 h. After this procedure, the purity of each IL was further checked by ^1^H nuclear magnetic resonance (NMR) spectra and found to be in accordance with the stated purity level provided by the suppliers (*cf.* Supplementary Figs 2–9). The water used was double distilled, passed through a reverse osmosis system, and further treated with a Milli-Q plus 185 apparatus. Cytochrome c and azocasein were purchased from Sigma-Aldrich.

### Phase diagrams and tie-lines

The solubility curves were determined through the cloud point titration method^22^ at 25, 35, 45 and 55 °C (±1 °C) and atmospheric pressure. Aqueous solutions of PPG at *circa* 80 wt % and aqueous solutions of the different ILs (with concentrations ranging from 50 wt % to 90 wt %) were prepared and used for the determination of the binodal curves. Repetitive drop wise addition of the aqueous PIL solution to the aqueous solutions of PPG was carried out until the detection of a cloudy (biphasic) solution, followed by the drop wise addition of ultrapure water until the finding of a monophasic region (clear and limpid solution). The ternary system compositions were determined by weight quantification within ±10^–4^ g.

The experimental binodal curves at 25 °C were fitted by equation (1)^49^.


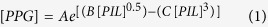


where [*PPG*] and [*PIL*] are the PPG and PIL weight fraction percentages, respectively, and *A*, *B*, and *C* are constants obtained by the regression of the experimental binodal data (see Supplementary Table 9).

The tie-lines were determined by a gravimetric method originally proposed by Merchuk *et al.*^49^ for polymer-based ABS, and later on applied by Rogers and co-workers^21^ to IL-based ABS. A ternary mixture composed of PPG + PIL + water at the biphasic region was gravimetrically prepared within ±10^-4^ g, vigorously agitated, and left to equilibrate for at least 12 h at (25 ±1) °C, aiming at a complete separation of the coexisting phases. After such time, both phases were carefully separated and individually weighed. Each tie-line was determined by the lever-arm rule through the relationship between the top phase composition and the overall system composition. Further details on the determination of the tie-lines and respective tie-line length can be found elsewhere^22^.

### pH and conductivity measurements

The pH values (± 0.02) of the PPG-rich and PIL-rich aqueous phases were measured at 25, 35, 45 and 55 °C ( ± 1 °C), and the electrical conductivity was measured at (25 ± 1) °C, using a Mettler Toledo SevenMultiTMdual pH/conductivity meter.

### Partitioning of cytochrome c and azocasein

Cytochrome c and azocasein were studied as representative examples of added-value proteins. Aqueous solutions of each biomolecule were prepared at the concentrations of 1, 2 and 3 g·L^−1^. Aiming at studying the possibility of moving from monophasic to biphasic regimes in PIL-based ABS, by temperature changes, an initial ternary mixture at the monophasic region was chosen based on the phase diagram of [N_11[2(N11)]0_][C_1_CO_2_]-based ABS: 6 wt % [N_11[2(N11)]0_][C_1_CO_2_] + 30 wt % PPG + 64 wt % aqueous solution of protein. The mixture was vigorously stirred and left to equilibrate at 45 °C for 12 h to achieve the complete partitioning of cytochrome c and azocasein between the two phases. After a careful separation of both phases, the quantification of cytochrome c and azocasein in each phase was carried out by UV-spectroscopy, using a BioTeck Synergy HT microplate reader, at a wavelength of 410 nm for cytochrome c and 342 nm for azocasein using calibration curves previously established. At least three individual samples were prepared and three samples of each phase were quantified in order to determine the average in the extractions efficiencies and respective standard deviations. Possible interferences of the PPG and PIL with the analytical method were controlled using blank control samples. The percentage extraction efficiency of cytochrome c and azocasein is defined as the percentage ratio between the amount of each protein in the PIL-aqueous-rich phase and that in the total mixture.

### Thermal stability of the protic ionic liquids

The PILs thermal stability, after the drying process and after being submitted at 55 °C for 12 h, as well as of the PIL- and PPG-rich phases of some systems after being submitted at 55 °C for 12 h, were evaluated by ^1^H and ^13^C NMR spectra using a Bruker Avance 300 at 300.13 MHz, with deuterium oxide (D_2_O) as solvent and trimethylsilyl propanoic acid (TSP) as internal reference.

### Proteins stability

The proteins stability in the PIL-rich phase after the extraction occurring at 45 °C was evaluated by Fourier Transform Infrared Spectroscopy (FTIR). The spectrum of each protein in a buffer solution at pH 7 and 25 °C and of the PIL-rich phases after the extraction of proteins at 45 °C were recorded. Blank control samples containing the same composition but without protein were always used. FTIR spectra were determined by a Perkin Elmer BX spectrometer operating in the attenuated total reflection (ATR) mode (equipped with a single horizontal Golden Gate ATR cell) with a resolution of 4 cm^−1^.

## Additional Information

**How to cite this article**: Passos, H. *et al.* Thermoreversible (Ionic-Liquid-Based) Aqueous Biphasic Systems. *Sci. Rep.* **6**, 20276; doi: 10.1038/srep20276 (2016).

## Supplementary Material

Supplementary Information
