# Supplementary material for: Thermoreversible (Ionic-Liquid-Based) Aqueous Biphasic Systems
Source: Sci Rep. 2016 Feb 4;6:20276. doi: 10.1038/srep20276 (PMC4740753; doi:10.1038/srep20276)
Supplement: Supplementary Information [file srep20276-s1.pdf]

Supporting Information

Thermoreversible (Ionic-Liquid-Based) Aqueous Biphasic Systems

Helena Passos, Andreia Luís, João A. P. Coutinho, Mara G. Freire*

CICECO - Aveiro Institute of Materials, Department of Chemistry, University of Aveiro, 3810-193 Aveiro, Portugal

*E-mail: maragfreire@ua.pt

Supplementary Figures

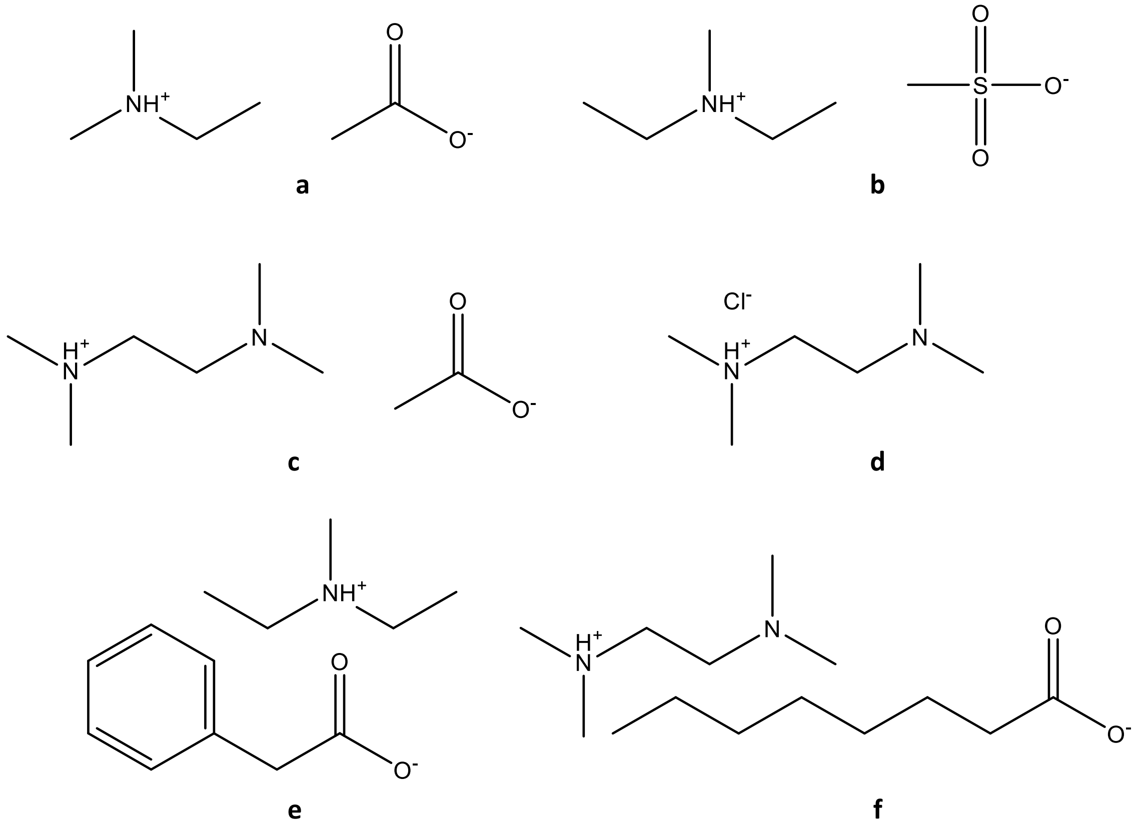

Supplementary Figure 1: Chemical structure of the PILs studied: (***a***) $[N_{1120}][C_1CO_2]$; (***b***) $[N_{1220}][C_1SO_3]$; (***c***) $[N_{11[2(N11)]0}][C_1CO_2]$; (***d***) $[N_{11[2(N11)]0}]Cl$; (***e***) $[N_{1120}][C_7H_7CO_2]$; (***f***) $[N_{11[2(N11)]0}][C_7CO_2]$.

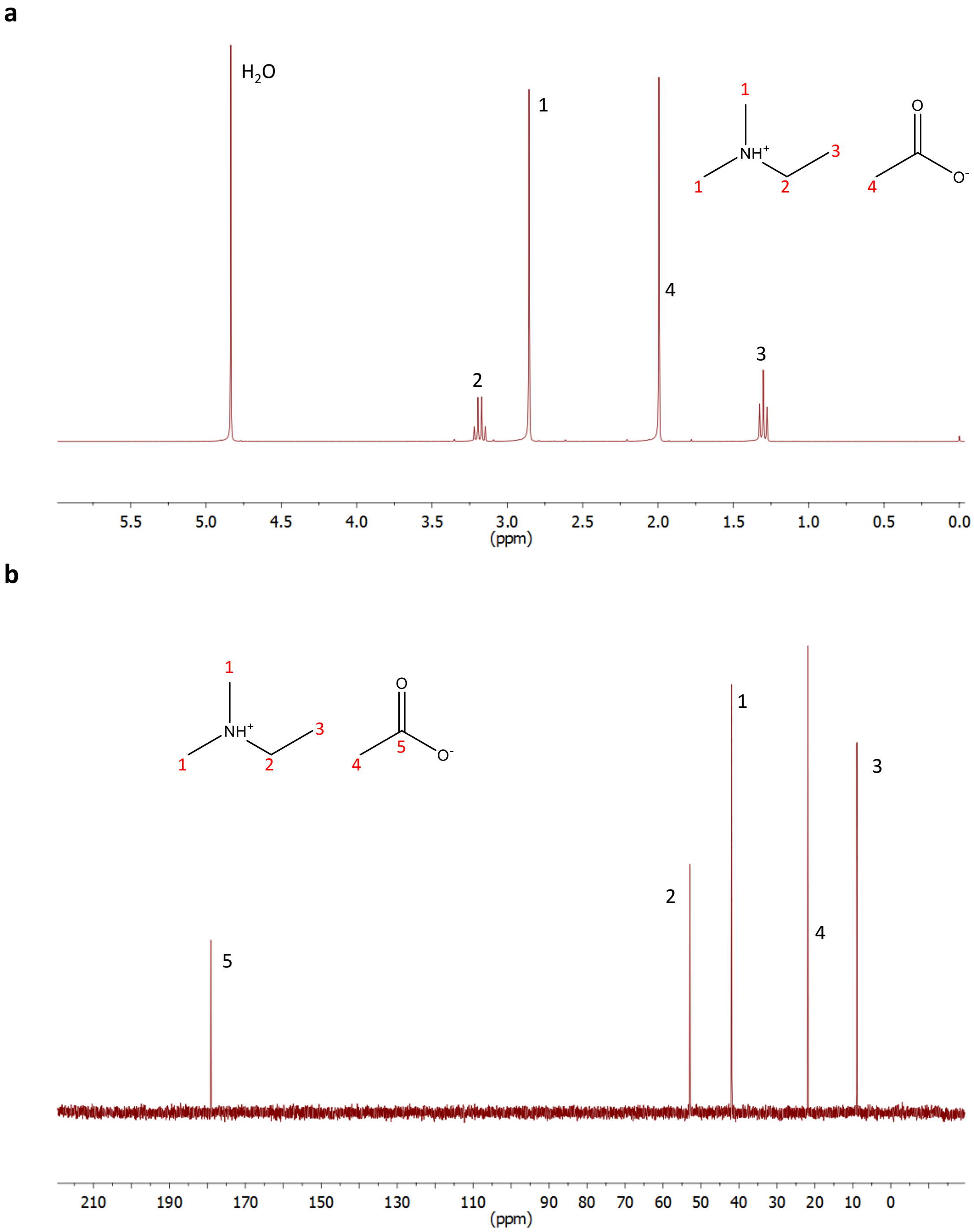

Supplementary Figure 2. NMR spectra of the purified $[N_{1120}][C_1CO_2]$ in D_2O. (***a***) ^{1}H NMR spectrum; (***b***) ^{13}C NMR spectrum. ^{1}H NMR (D_2O, 300 MHz, [ppm]): δ 3.22-3.15 (m, 2H, $NC\underline{H}_2CH_3$), 2.86 (s, 6H, $N(C\underline{H}_3)_2$), 1.99 (s, 3H, $C\underline{H}_3CO_2$), 1.33-1.28 (m, 3H, $NCH_2C\underline{H}_3$). ^{13}C NMR (D_2O, 75.47 MHz, [ppm]): δ 179.1 ($CH_3\underline{C}O_2$), 52.9 ($N\underline{C}H_2CH_3$), 42.0 ($N(\underline{C}H_3)_2$), 21.8 ($\underline{C}H_3CO_2$), 9.1 ($NCH_2\underline{C}H_3$).

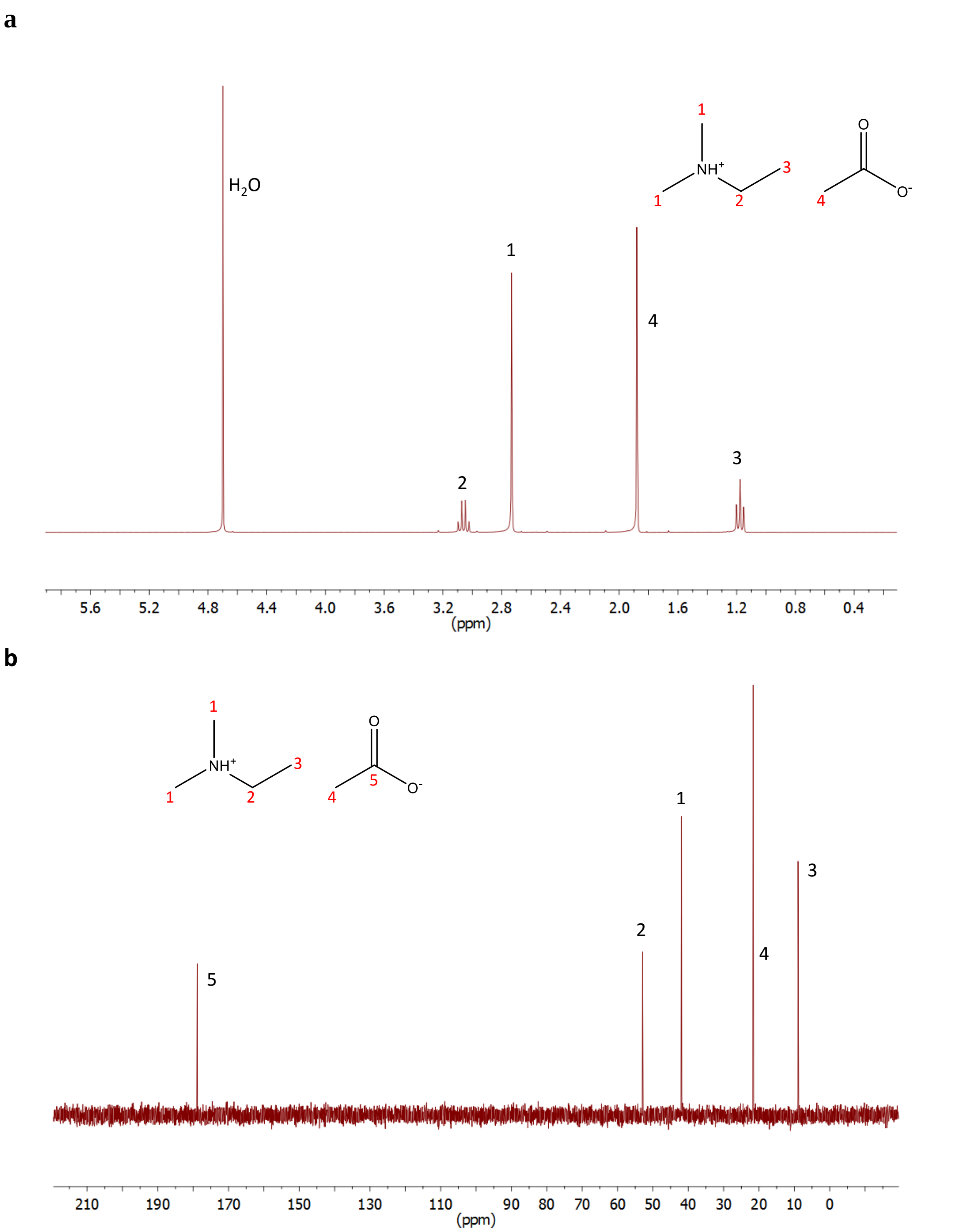

Supplementary Figure 3. NMR spectra of the purified $[N_{1120}][C_1CO_2]$ in D_2O, and after 12 h at 55 ºC. (***a***) ^1H NMR spectrum; (***b***) ^{13}C NMR spectrum. ^1H NMR (D_2O, 300 MHz, [ppm]): δ 3.10-3.02 (m, 2H, $NC\underline{H}_2CH_3$), 2.73 (s, 6H, $N(C\underline{H}_3)_2$), 1.88 (s, 3H, $C\underline{H}_3CO_2$), 1.20-1.15 (m, 3H, $NCH_2C\underline{H}_3$). ^{13}C NMR (D_2O, 75.47 MHz, [ppm]): δ 178.8 ($CH_3\underline{C}O_2$), 52.9 ($N\underline{C}H_2CH_3$), 41.9 ($N(\underline{C}H_3)_2$), 21.6 ($\underline{C}H_3CO_2$), 8.9 ($NCH_2\underline{C}H_3$).

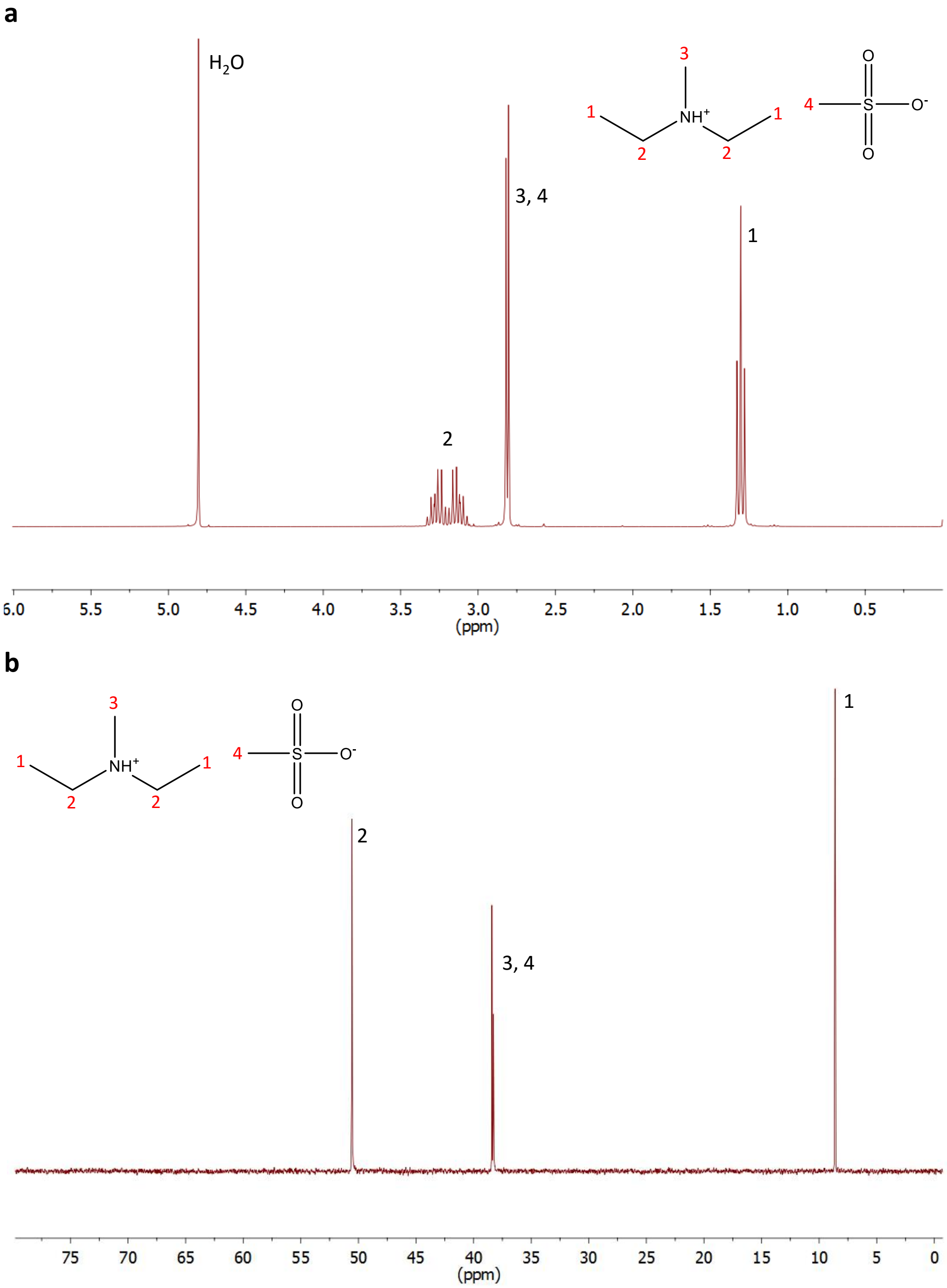

Supplementary Figure 4. NMR spectra of the purified [N_{1220}][C_1SO_3] in D_2O. (***a***) ^{1}H NMR spectrum; (***b***) ^{13}C NMR spectrum. ^{1}H NMR (D_2O, 300 MHz, [ppm]): δ 3.33-3.07 (m, 4H, $N(C\underline{H}_2CH_3)_2$), 2.82 (s, 3H, $NC\underline{H}_3$), 2.80 (s, 3H, $SC\underline{H}_3$) 1.33-1.28 (m, 6H, $N(CH_2C\underline{H}_3)_2$). ^{13}C NMR ($D_2O$, 75.47 MHz, [ppm]): δ 50.6 ($N(\underline{C}H_2CH_3)_2$), 38.4 ($N\underline{C}H_3$), 38.3 ($S\underline{C}H_3$), 8.6 ($N(CH_2\underline{C}H_3)_2$).

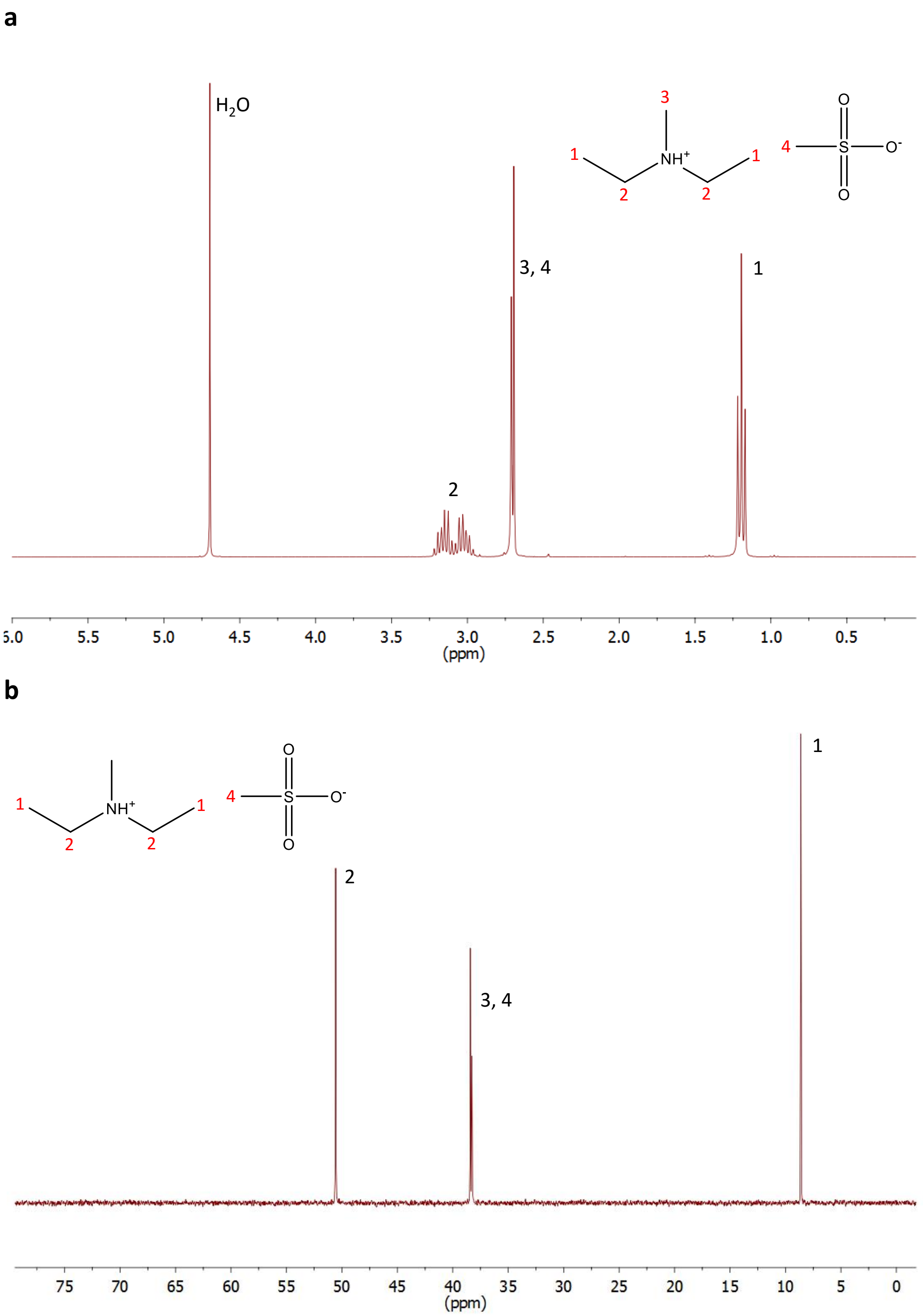

Supplementary Figure 5. NMR spectra of the purified $[N_{1220}][C_1SO_3]$ in D_2O, and after 12 h at 55 ºC. (***a***) ^{1}H NMR spectrum; (***b***) ^{13}C NMR spectrum. ^{1}H NMR (D_2O, 300 MHz, [ppm]): δ 3.22-2.96 (m, 4H, $N(C\underline{H}_2CH_3)_2$), 2.71 (s, 3H, $NC\underline{H}_3$), 2.69 (s, 3H, $SC\underline{H}_3$) 1.22-1.17 (m, 6H, $N(CH_2C\underline{H}_3)_2$). ^{13}C NMR ($D_2O$, 75.47 MHz, [ppm]): δ 50.6 ($N(\underline{C}H_2CH_3)_2$), 38.4 ($N\underline{C}H_3$), 38.3 ($S\underline{C}H_3$), 8.6 ($N(CH_2\underline{C}H_3)_2$).

a

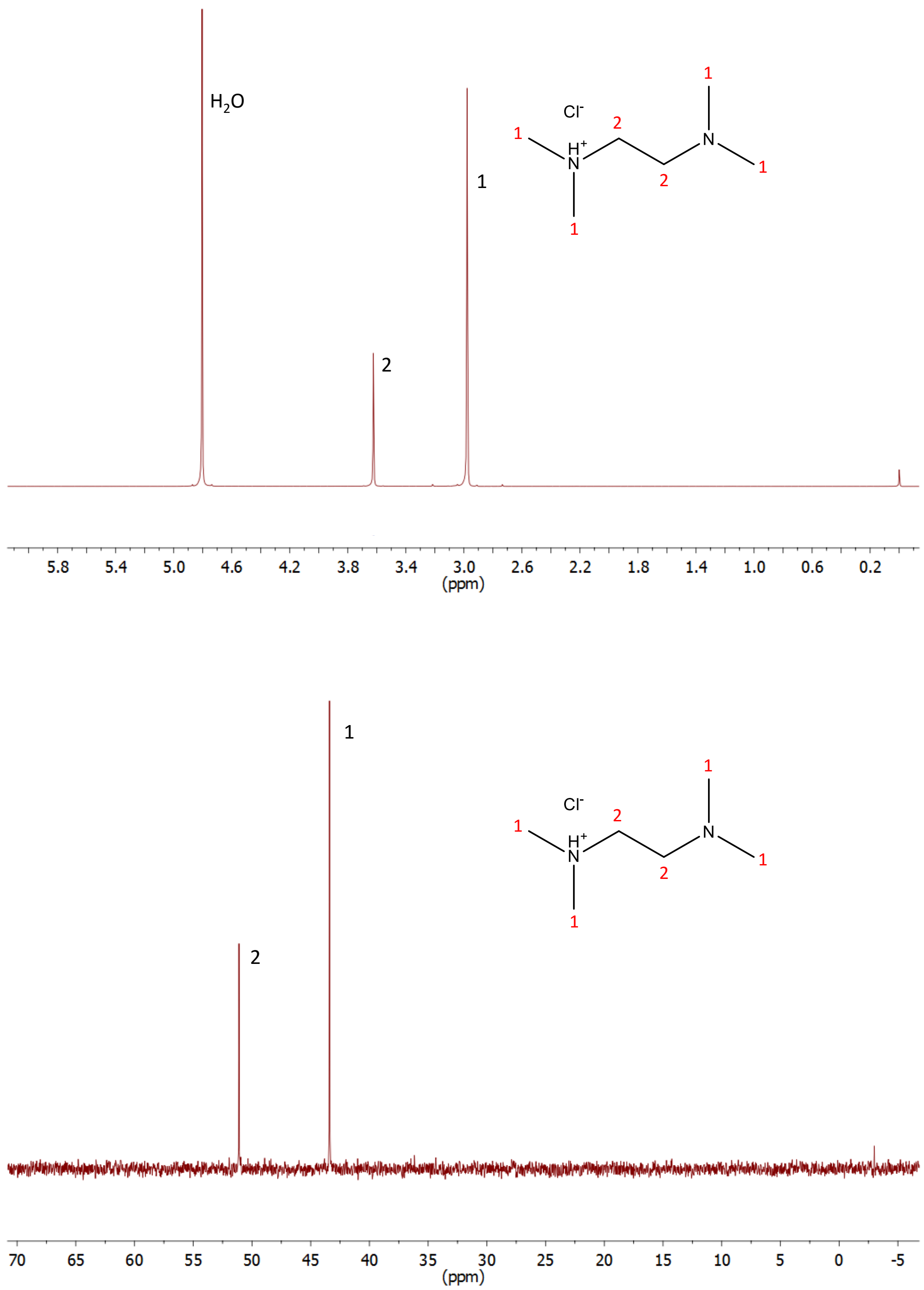

Supplementary Figure 6. NMR spectra of the purified $[N_{11[2(N11)0]}]Cl$ in D_2O. (***a***) ^{1}H NMR spectrum; (***b***) ^{13}C NMR spectrum. ^{1}H NMR (D_2O, 300 MHz, [ppm]): δ 3.62 (s, 4H, $N(C\underline{H}_2)_2$), 2.98 (s, 12H, $2N(C\underline{H}_3)_2$). ^{13}C NMR (D_2O, 75.47 MHz, [ppm]): δ 51.1 ($N(\underline{C}H_2)_2$), 43.4 ($N(\underline{C}H_3)_2$).

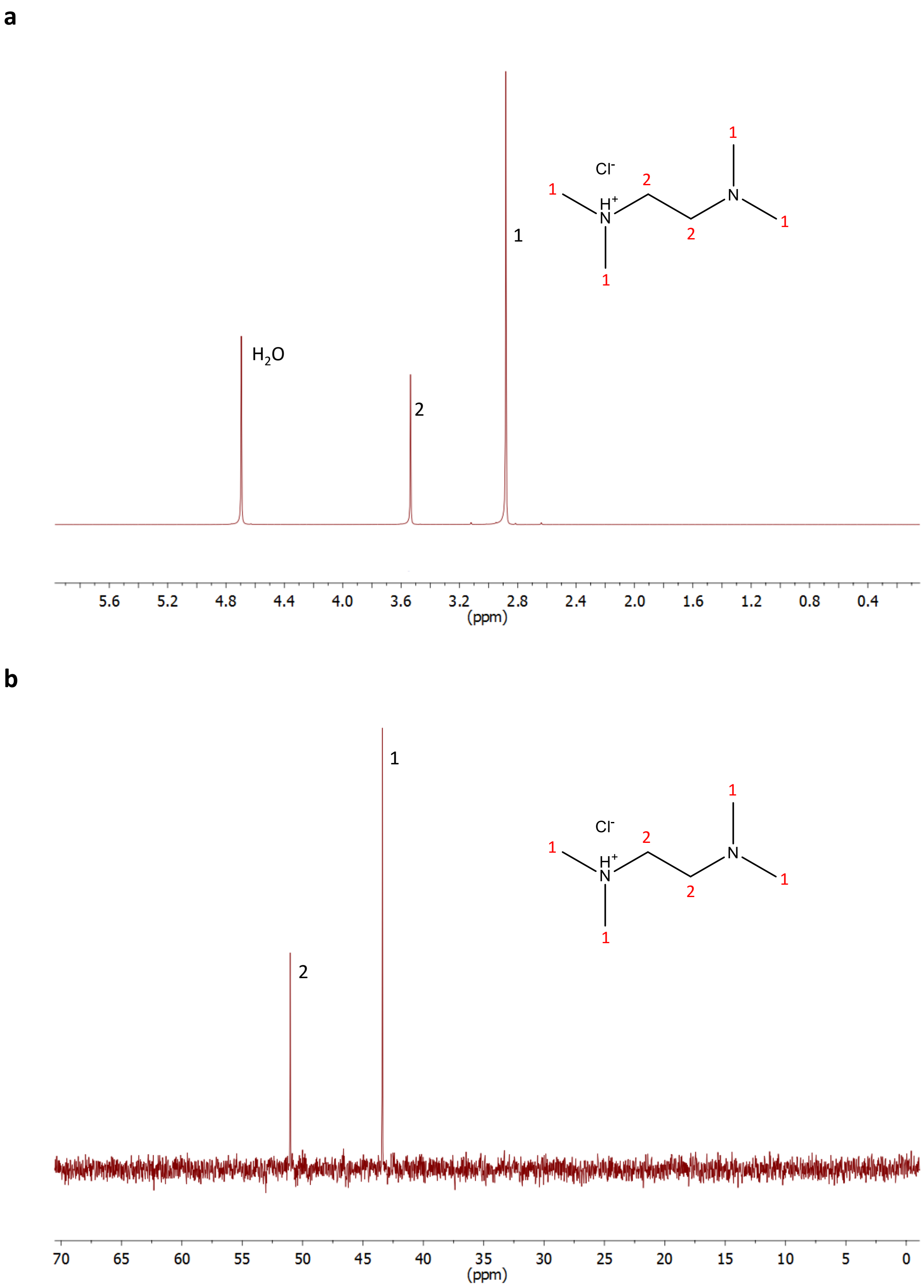

Supplementary Figure 7. NMR spectra of the purified $[N_{11[2(N11)0]}]Cl$ in D_2O, and after 12 h at 55 ºC. (***a***) ^1H NMR spectrum; (***b***) ^{13}C NMR spectrum. ^1H NMR (D_2O, 300 MHz, [ppm]): δ 3.54 (s, 4H, $N(C\underline{H}_2)_2$), 2.88 (s, 12H, $2[N(C\underline{H}_3)_2]$). ^{13}C NMR (D_2O, 75.47 MHz, [ppm]): δ 51.0 ($N(\underline{C}H_2)_2$), 43.4 ($N(\underline{C}H_3)_2$).

a

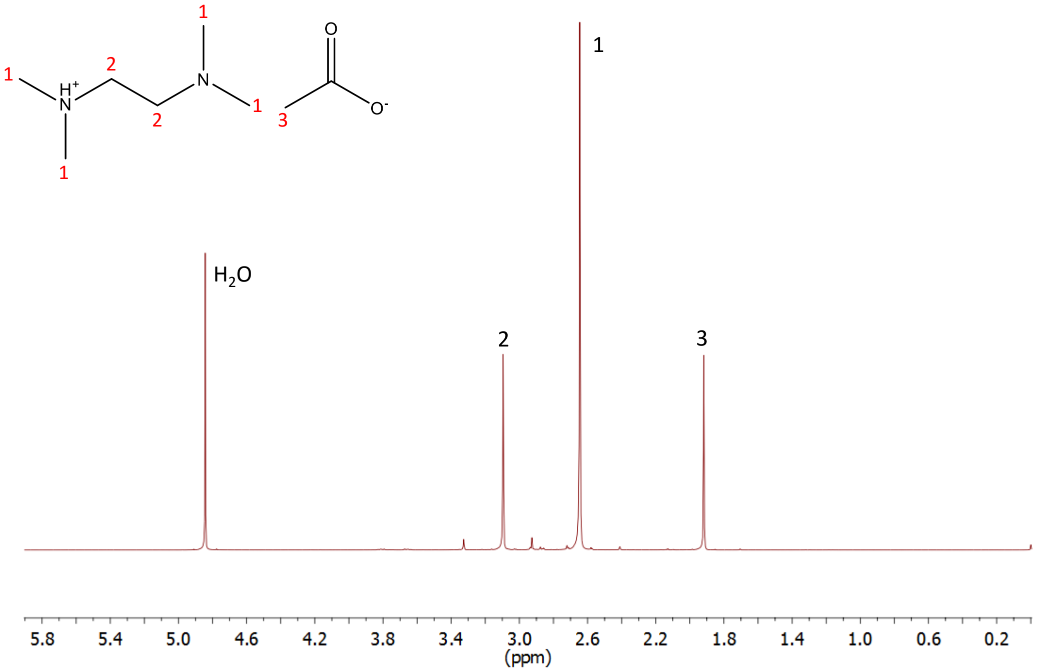

b

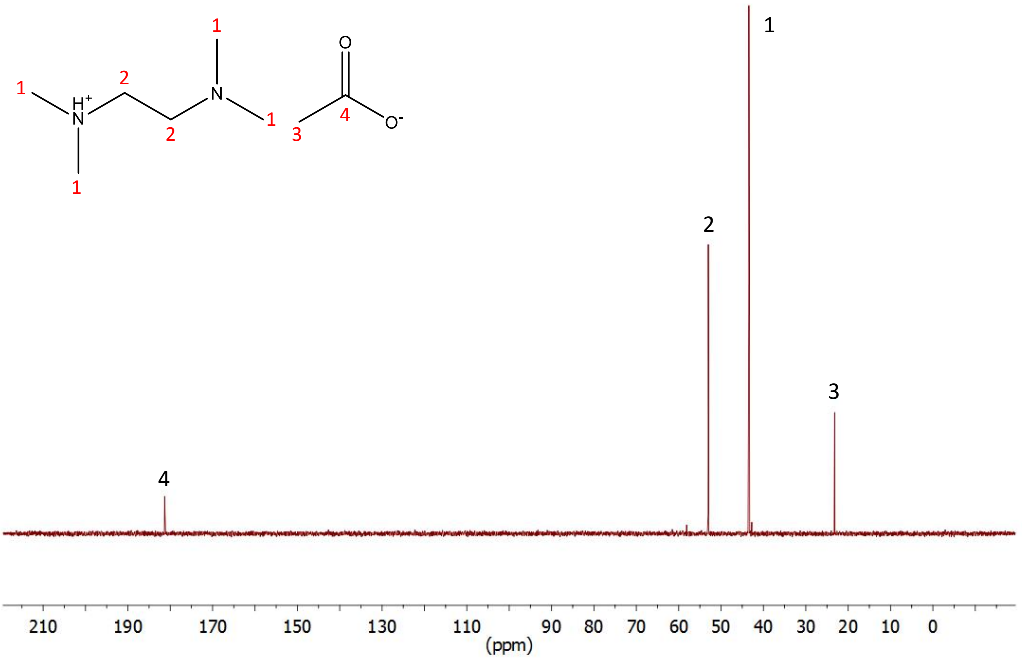

Supplementary Figure 8. NMR spectra of the purified $[N_{11[2(N11)0]}][C_1CO_2]$ in D_2O. (***a***) ^1H NMR spectrum; (***b***) ^{13}C NMR spectrum. ^1H NMR (D_2O, 300 MHz, [ppm]): δ 3.10 (s, 4H, $N(C\underline{H}_2)_2$), 2.65 (s, 12H, $2[N(C\underline{H}_3)_2]$), 1.92 (s, 3H, $C\underline{H}_3CO_2$) ^{13}C NMR (D_2O, 75.47 MHz, [ppm]): δ 181.3 ($CH_3\underline{C}O_2$), 53.0 ($N(\underline{C}H_2)_2$), 43.4 ($N(\underline{C}H_3)_2$), 23.2 ($\underline{C}H_3CO_2$).

a

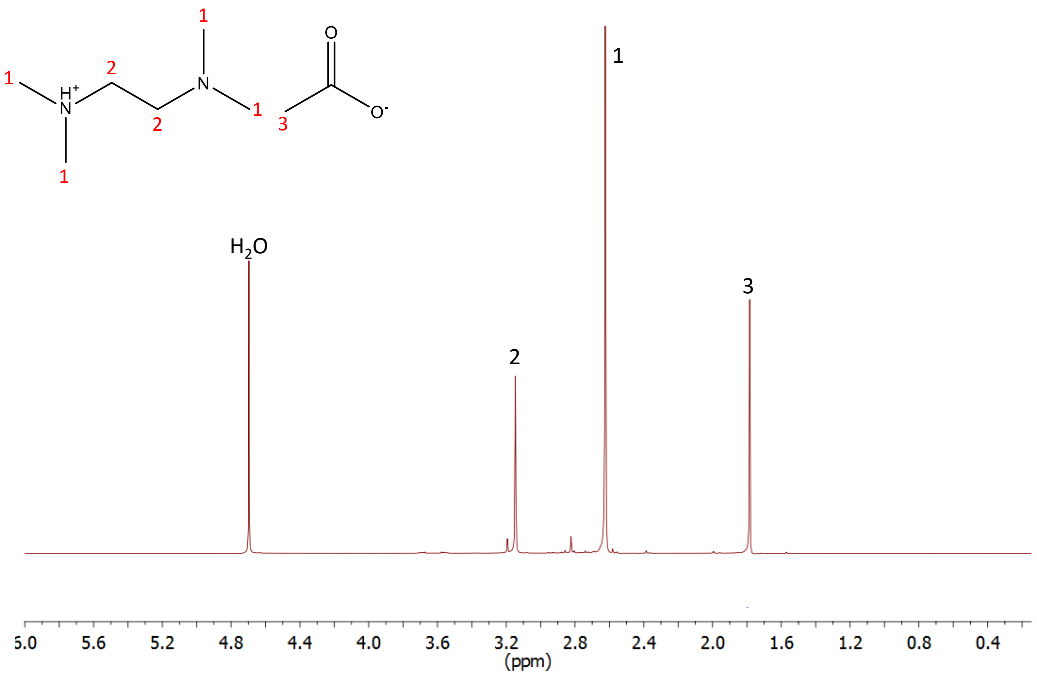

b

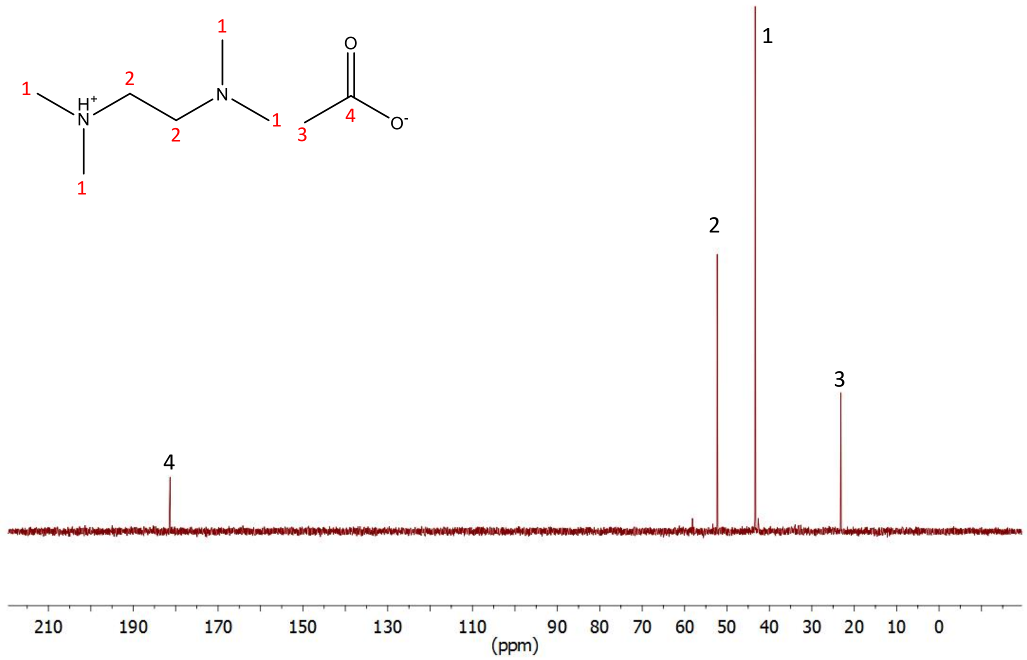

Supplementary Figure 9. NMR spectra of the purified $[N_{11[2(N11)0]}][C_1CO_2]$ in D_2O, and after 12 h at 55 °C. (***a***) ^{1}H NMR spectrum; (***b***) ^{13}C NMR spectrum. ^{1}H NMR (D_2O, 300 MHz, [ppm]): δ 3.15 (s, 4H, $N(C\underline{H}_2)_2$), 2.62 (s, 12H, $2[N(C\underline{H}_3)_2]$), 1.78 (s, 3H, $C\underline{H}_3CO_2$) ^{13}C NMR (D_2O, 75.47 MHz, [ppm]): δ 181.3 ($CH_3\underline{C}O_2$), 52.3 ($N(\underline{C}H_2)_2$), 43.4 ($N(\underline{C}H_3)_2$), 23.1 ($\underline{C}H_3CO_2$).

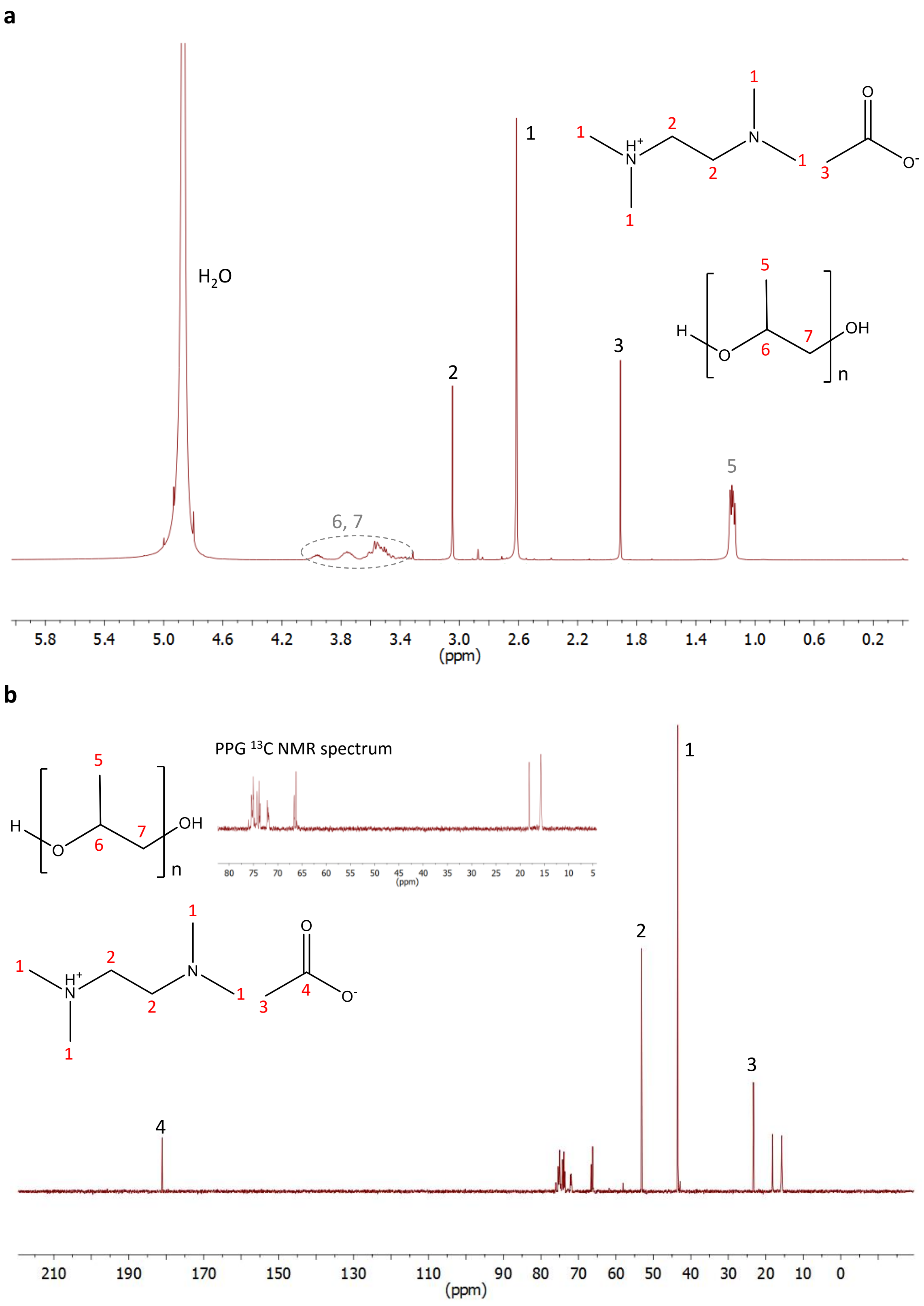

Supplementary Figure 10. NMR spectra of the PIL-rich phase of the ABS composed of 6 wt % $[N_{11[2(N11)]0}][C_1CO_2]$ + 30 wt % PPG + 54 wt % H_2O ABS in D_2O, and after 12 h at 55 ºC. (***a***) ^{1}H NMR spectrum; (***b***) ^{13}C NMR spectrum. ^{1}H NMR (D_2O, 300 MHz,

[ppm]): δ 4.02-3.34 (m, (*n*+2*n*)H, $n[C\underline{H}C\underline{H}_2]$), 3.05 (s, 4H, $N(C\underline{H}_2)_2$), 2.61 (s, 12H, $2[N(C\underline{H}_3)_2]$), 1.91 (s, 3H, $C\underline{H}_3CO_2$), 1.17-1.14 (m, 3*n*H, $n[CHC\underline{H}_3]$). ^{13}C NMR (D_2O, 75.47 MHz, [ppm]): δ 181.1 ($CH_3\underline{C}O_2$), 76.0-66.0 (PPG) 53.1 ($N(\underline{C}H_2)_2$), 43.5 ($N(\underline{C}H_3)_2$), 23.3 ($\underline{C}H_3CO_2$), 18.2 (PPG), 15.8 (PPG).

a

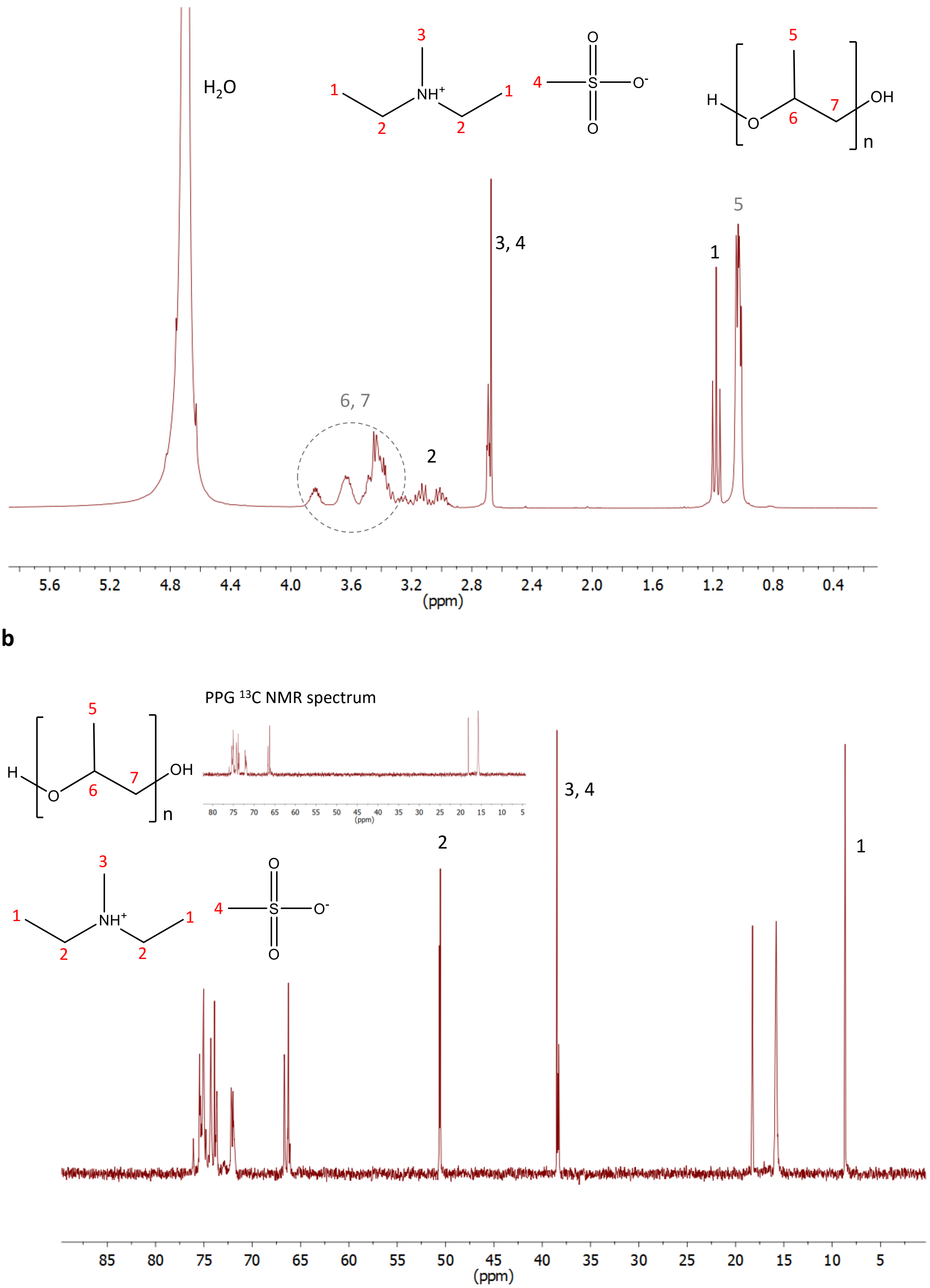

Supplementary Figure 11. NMR spectra of the PIL-rich phase of the ABS composed of 6 wt % $[N_{1220}][C_1SO_3]$ + 30 wt % PPG + 54 wt % H_2O ABS in D_2O, and after 12 h at 55

°C. (***a***) ^{1}H NMR spectrum. (***b***) ^{13}C NMR spectrum. ^{1}H NMR (D_2O, 300 MHz, [ppm]): δ 3.87-3.24 (m, (*n*+2*n*)H, $n[C\underline{H}C\underline{H}_2]$), 3.17-2.97 (m, 4H, $N(C\underline{H}_2CH_3)_2$), 2.69 (s, 3H, $NC\underline{H}_3$), 2.67 (s, 3H, $SC\underline{H}_3$), 1.20-1.15 (m, 6H, $N(CH_2C\underline{H}_3)_2$), 1.05-1.01 (m, 3*n*H, $n[CHC\underline{H}_3]$). ^{13}C NMR (D_2O, 75.47 MHz, [ppm]): δ 76.1-66.1 (PPG), 50.5 ($N(\underline{C}H_2CH_3)_2$), 38.4 ($N\underline{C}H_3$), 38.3 ($S\underline{C}H_3$), 18.2 (PPG), 15.8 (PPG) 8.7 ($N(CH_2\underline{C}H_3)_2$).

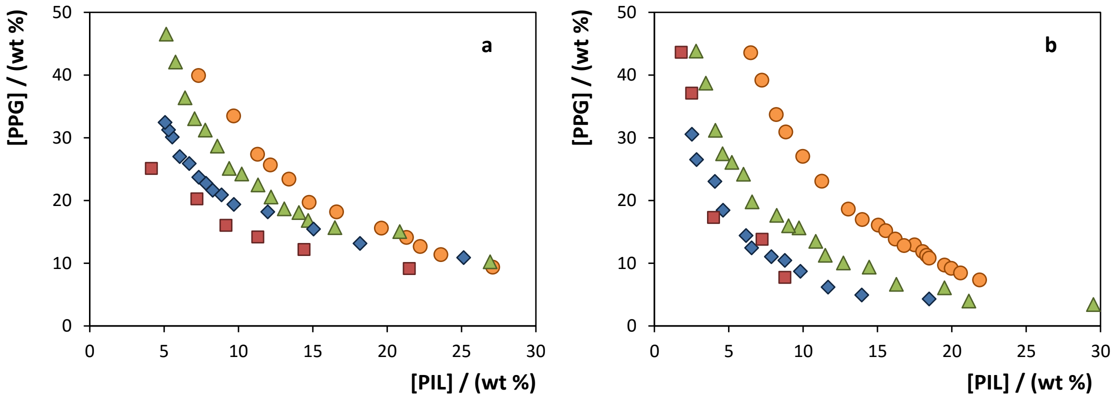

Supplementary Figure 12. Evaluation of the temperature effect in the phase diagrams behavior of ABS composed of PIL + PPG + H_2O at 25 ºC (orange circle), 35 ºC (green triangles), 45 ºC (blue diamonds) and 55 ºC (red squares). (***a***) $[N_{1220}][C_1SO_3]$. (***b***) $[N_{11[2(N11)]0}]Cl$.

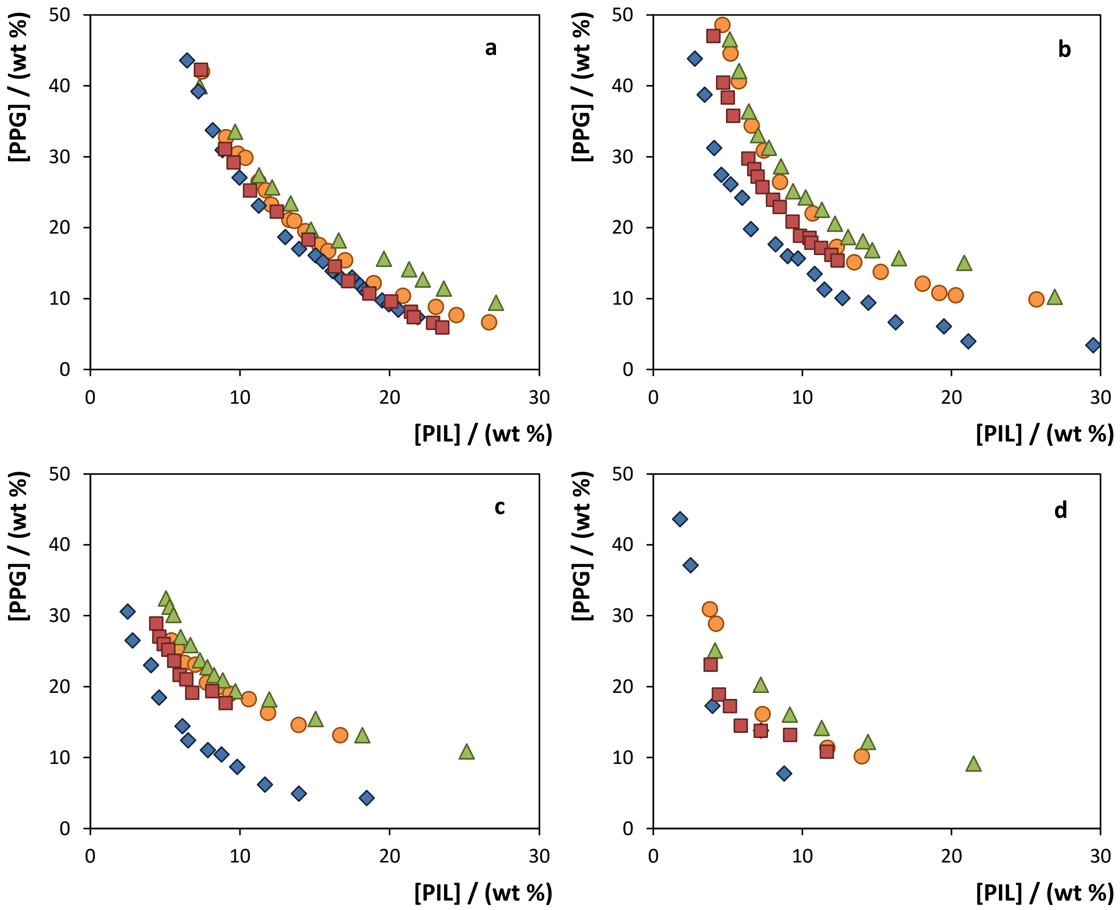

Supplementary Figure 13. Evaluation of the IL effect in the phase diagrams behavior of ABS composed of PIL + PPG + H_2O: $[N_{1120}][C_1CO_2]$ (orange circles), $[N_{1220}][C_1SO_3]$ (green triangles), $[N_{11[2(N11)]0}]Cl$ (blue diamonds), $[N_{11[2(N11)]0}][C_1CO_2]$ (red squares). (***a***) 25 ºC. (***b***) 35 ºC. (***c***) 45 ºC. (***d***) 55 ºC.

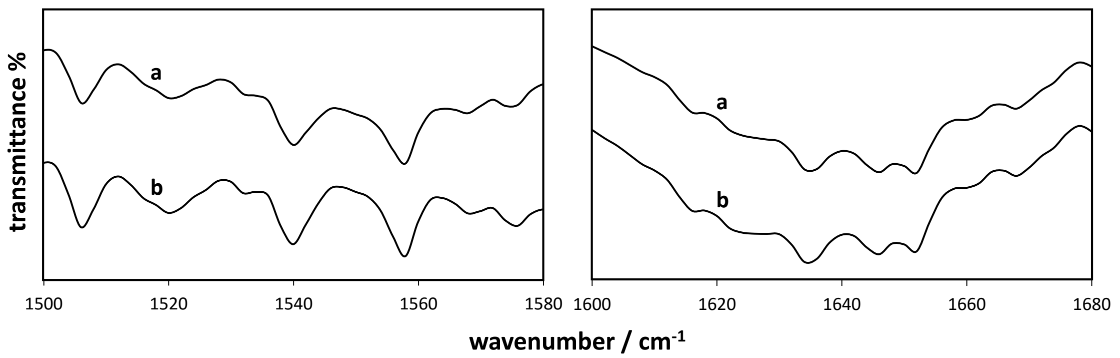

Supplementary Figure 14. FTIR spectra of (***a***) PIL-rich phase of ABS composed of 6 wt % of $[N_{11[2(N11)]0}][C_1CO_2]$ + 30 wt % of PPG + 64 wt % H_2O with azocasein at 45 ºC and (***b***) azocasein in a buffered aqueous solution at pH 7 (PBS) at 25 ºC.

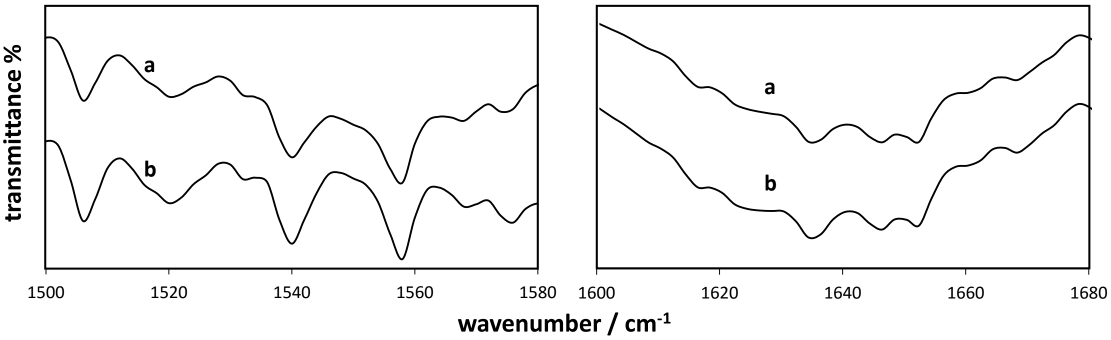

Supplementary Figure 15. FTIR spectra of (***a***) PIL-rich phase of ABS composed of 6 wt % of [$N_{11[2(N11)]0}$][C_1CO_2] + 30 wt % of PPG + 64 wt % H_2O with cytochrome c at 45 ºC and (***b***) cytochrome c in a buffered aqueous solution at pH 7 (PBS) at 25 ºC.

Supplementary Tables

Supplementary Table 1. Experimental weight fraction data for the system composed of PPG (1) + PIL (2) + H_2O (3) at 25 ºC and atmospheric pressure.

$[N_{1120}][C_1CO_2]$		$[N_{1220}][C_1SO_3]$		$[N_{11[2(N11)]0}]Cl$		$[N_{11[2(N11)]0}][C_1CO_2]$	
100 w_1	100 w_2	100 w_1	100 w_2	100 w_1	100 w_2	100 w_1	100 w_2
82.53	3.55	51.93	5.39	43.56	6.47	42.25	7.39
50.09	6.61	39.95	7.31	39.21	7.21	31.08	9.00
42.00	7.45	33.50	9.67	33.71	8.17	29.19	9.57
32.75	9.07	27.37	11.28	30.97	8.83	25.25	10.67
30.44	9.85	25.68	12.14	27.05	9.96	22.25	12.45
29.86	10.37	23.40	13.39	23.09	11.25	18.32	14.57
26.45	11.24	19.73	14.74	18.66	13.02	14.51	16.34
25.28	11.72	18.19	16.60	16.98	13.96	12.47	17.23
23.22	12.08	15.61	19.62	16.10	15.04	10.72	18.64
21.08	13.30	14.13	21.31	15.21	15.55	9.58	20.10
20.95	13.63	12.66	22.21	13.86	16.19	8.11	21.42
19.48	14.36	11.39	23.62	12.95	17.49	7.37	21.61
17.54	15.29	9.39	27.10	12.83	16.78	6.56	22.89
16.70	15.89			11.88	18.02	5.92	23.52
15.40	17.03			11.27	18.29		
12.16	18.93			10.83	18.47		
10.37	20.88			9.73	19.49		
8.83	23.09			9.18	19.95		
7.67	24.46			8.44	20.57		
6.64	26.64			7.35	21.86		

Supplementary Table 2. Experimental weight fraction data for the system composed of PPG (1) + PIL (2) + H_2O (3) at 35 ºC and atmospheric pressure.

$[N_{1120}][C_1CO_2]$		$[N_{1220}][C_1SO_3]$		$[N_{11[2(N11)]0}]Cl$		$[N_{11[2(N11)]0}][C_1CO_2]$	
100 w_1	100 w_2	100 w_1	100 w_2	100 w_1	100 w_2	100 w_1	100 w_2
56.54	3.63	53.49	4.12	43.83	2.79	47.01	4.03
48.58	4.64	46.54	5.14	38.72	3.44	40.43	4.69
44.56	5.20	42.07	5.77	31.21	4.09	38.34	5.01
40.63	5.75	36.38	6.41	27.46	4.57	35.78	5.36
34.38	6.60	33.03	7.04	26.10	5.20	29.75	6.37
30.84	7.41	31.24	7.76	24.211	5.97	28.23	6.77
26.44	8.51	28.66	8.57	19.80	6.56	27.19	6.99
22.00	10.70	25.12	9.37	17.65	8.21	25.69	7.33
17.28	12.31	24.25	10.22	15.97	9.01	23.94	8.04
15.10	13.49	22.50	11.31	15.65	9.71	22.89	8.49
13.77	15.26	20.57	12.18	13.48	10.83	20.82	9.35
12.09	18.07	18.67	13.08	11.27	11.48	18.83	9.85
10.79	19.19	18.07	14.06	10.06	12.69	18.55	10.48
10.44	20.29	16.81	14.68	9.39	14.43	17.86	10.60
9.89	25.71	15.67	16.48	6.63	16.26	17.13	11.26
		15.03	20.85	6.08	19.50	16.14	11.94
		10.25	26.92	3.97	21.13	15.35	12.36
				3.43	29.51		

Supplementary Table 3. Experimental weight fraction data for the system composed of PPG (1) + PIL (2) + H_2O (3) at 45 ºC and atmospheric pressure.

$[N_{1120}][C_1CO_2]$		$[N_{1220}][C_1SO_3]$		$[N_{11[2(N11)]0}]Cl$		$[N_{11[2(N11)]0}][C_1CO_2]$	
100 w_1	100 w_2	100 w_1	100 w_2	100 w_1	100 w_2	100 w_1	100 w_2
26.52	5.42	32.47	5.06	30.58	2.50	28.93	4.40
25.41	5.79	31.28	5.30	26.53	2.82	27.07	4.61
23.37	6.29	30.12	5.55	23.06	4.05	26.04	4.89
23.12	7.02	27.00	6.04	18.46	4.60	25.24	5.21
20.57	7.78	25.88	6.69	14.43	6.16	23.65	5.60
19.96	8.53	23.73	7.33	12.45	6.53	21.66	5.96
19.03	9.35	22.76	7.82	11.05	7.86	21.04	6.40
18.27	10.59	21.64	8.26	10.46	8.76	19.16	6.81
16.32	11.86	20.91	8.86	8.71	9.82	19.36	8.14
14.63	13.92	19.38	9.68	6.20	11.66	17.70	9.05
13.16	16.70	18.21	11.96	4.94	13.94		
		15.45	15.04	4.32	18.46		
		13.17	18.17	3.34	30.05		
		10.89	25.15				

Supplementary Table 4. Experimental weight fraction data for the system composed of PPG (1) + PIL (2) + H_2O (3) at 55 ºC and atmospheric pressure.

$[N_{1120}][C_1CO_2]$		$[N_{1220}][C_1SO_3]$		$[N_{11[2(N11)]0}]Cl$		$[N_{11[2(N11)]0}][C_1CO_2]$	
100 w_1	100 w_2	100 w_1	100 w_2	100 w_1	100 w_2	100 w_1	100 w_2
30.92	3.78	25.11	4.14	43.62	1.79	23.12	3.84
28.90	4.20	20.29	7.21	37.12	2.50	18.91	4.40
16.15	7.33	16.06	9.15	17.30	3.96	17.28	5.14
11.38	11.67	14.19	11.30	13.84	7.22	14.50	5.86
10.21	13.99	12.21	14.40	7.75	8.77	13.80	7.20
		9.17	21.48			13.21	9.17
						10.86	11.64

Supplementary Table 5. Experimental data for tie-lines and tie-line length (TLL) of PILs + PPG ABS at 25 ºC, and pH of the coexisting phases.

PIL	Weight fraction composition / wt %								TLL
	**[*PIL*]$_{PIL}$**	**[*PPG*]$_{IL}$**	**pH$_{PIL}$**	**[*PIL*]$_{M}$**	**[*PPG*]$_{M}$**	**[*PIL*]$_{PPG}$**	**[*PPG*]$_{PPG}$**	**pH$_{PPG}$**	
$[N_{1120}][C_1CO_2]$	22.98	8.10	6.00	11.13	49.94	4.00	75.10	5.87	69.63
	26.13	6.02	5.98	9.96	59.65	3.63	80.62	5.87	77.91
$[N_{1220}][C_1SO_3]$	22.82	11.63	2.88	14.94	40.22	1.77	88.03	3.43	79.25
	32.98	5.36	2.32	20.10	40.07	1.68	89.72	2.21	89.97
$[N_{11[2(N11)]0}]Cl$	16.97	12.67	3.96	7.92	54.61	3.18	76.55	3.78	65.35
	23.66	6.62	3.97	10.75	54.62	2.73	84.43	3.90	80.57
$[N_{11[2(N11)]0}][C_1CO_2]$	18.86	10.54	7.93	10.15	50.27	3.31	81.48	7.95	72.62
	27.16	4.32	7.92	12.97	50.14	3.32	81.32	7.96	80.61

Supplementary Table 6. Electrical conductivity (κ) data for the PIL-rich and PPG-rich phases of the systems composed of PPG + PIL + H_2O (1:1000 (v:v) dilution).

PIL	[*PIL*]$_M$	[*PPG*]$_M$	10^{-3} κ / (μS·cm^{-1})	
			**Top phase**	**Bottom phase**
**25 ºC**				
$[N_{1120}][C_1CO_2]$	11.13	49.94	118.1	23.5
	9.96	59.65	134.8	21.4
$[N_{1220}][C_1SO_3]$	14.94	40.22	69.0	105.1
	20.10	40.07	64.2	156.8
$[N_{11[2(N11)0}]Cl$	7.92	54.61	6.44	308.0
	10.75	54.62	3.73	421.0
$[N_{11[2(N11)]0}][C_1CO_2]$	10.15	50.27	89.2	9.76
	12.97	50.14	110.8	9.36
**45 ºC**				
$[N_{11[2(N11)]0}][C_1CO_2]$	6.00	30.00	9.09	64.2

Supplementary Table 7. Extraction efficiencies of cytochrome c (EE_{Cyt}%) and azocasein (EE_{Azo}%) for the PIL-rich phase, obtained in ternary mixture composed of 6 wt % of $[N_{11[2(N11)]0}][C_1CO_2]$ + 30 wt % of PPG + 64 wt % of an aqueous solution containing the proteins at 1, 2 and 3 $g \cdot L^{-1}$, and equilibrated at 45 ºC.

	EE_{Azo}% ± 0.9	EE_{Cyt}% ± 0.3
**1 $g \cdot L^{-1}$**	95.36	99.85
**2 $g \cdot L^{-1}$**	95.56	100.00
**3 $g \cdot L^{-1}$**	95.30	100.00

Supplementary Table 8. pH data for the PIL-rich and PPG-rich phases of the systems composed of PPG + PIL + H_2O at 25, 35, 45 and 55 ºC for the following mixtures points: 9.96 wt % [N_{1120}][C_1CO_2] + 59.65 wt % PPG + 30.39 wt % H_2O; 20.10 wt % [N_{1220}][C_1SO_3] + 40.07 wt % PPG + 39.83 wt % H_2O; 10.75 wt % [$N_{11[2(N11)]0}$]Cl + 54.62 wt % PPG + 34.63 wt % H_2O; and 15.28 wt % [$N_{11[2(N11)]0}$][C_1CO_2] + 59.65 wt % PPG + 25.07 wt % H_2O.

PIL	25 °C		35 °C		45 °C		55 °C	
	pH_{PIL}	pH_{PPG}	pH_{PIL}	pH_{PPG}	pH_{PIL}	pH_{PPG}	pH_{PIL}	pH_{PPG}
[N_{1120}][C_1CO_2]	5.98	5.87	6.30	6.26	5.98	5.85	6.03	6.19
[N_{1220}][C_1SO_3]	2.32	2.21	2.64	2.93	2.42	2.31	2.14	2.77
[$N_{11[2(N11)]0}$]Cl	3.97	3.90	3.73	3.65	3.92	3.85	3.52	3.61
[$N_{11[2(N11)]0}$][C_1CO_2]	7.74	7.83	8.07	7.96	7.84	7.77	8.02	8.00

Supplementary Table 9. Correlation parameters used to describe the experimental binodal data at 25 ºC by equation (1).

IL	$A \pm \sigma$	$B \pm \sigma$	$10^5 C \pm \sigma$	R^2
$[N_{1120}][C_1CO_2]$	339.3 ± 11.8	-0.754 ± 0.014	1.00 ± 0.59	0.9976
$[N_{1220}][C_1SO_3]$	183.5 ± 14.6	-0.553 ± 0.030	1.00 ± 0.68	0.9944
$[N_{11[2(N11)]0}]Cl$	290.0 ± 17.9	-0.747 ± 0.023	1.12 ± 0.65	0.9985
$[N_{11[2(N11)]0}][C_1CO_2]$	327.5 ± 63.0	-0.765 ± 0.067	1.71 ± 1.49	0.9928
